# Association of eHealth Literacy With Cognitive Function, Frailty, and Functional Status in Hospitalized Geriatric Patients: Cross-Sectional Observational Study

**DOI:** 10.2196/92045

**Published:** 2026-11-09

**Authors:** Chantal Flemm, Rainer Wirth, Sarah El Bataioui, Diana Daubert, Ida Haussleiter, Matthias Tristan Hermenau, Jan Dieris-Hirche, Judith Frederica Kuhlmann, Katharina Kunde, Ulrike Trampisch, Bianca Ueberberg, Horst Christian Vollmar, Theresa Sophie Busse

**Affiliations:** 1 Digital Health, Department of Human Medicine, Faculty of Health, Witten/Herdecke University, Alfred-Herrhausen-Straße 50, Witten, North Rhine-Westphalia, 58455, Germany, 49 23234995970; 2 Department of Geriatric Medicine, Marien Hospital Herne, University Hospitals of the Ruhr-University of Bochum, Herne, North Rhine-Westphalia, Germany; 3 Department of Psychosomatic Medicine and Psychotherapy, LWL-Universitätsklinikum Bochum, Bochum, North Rhine-Westphalia, Germany; 4 Institute of General Practice and Family Medicine (AM RUB), Medical Faculty, Ruhr University Bochum, Bochum, North Rhine-Westphalia, Germany; 5 Department of Psychiatry and Psychotherapy, LWL-Universitätsklinikum Bochum, Bochum, North Rhine-Westphalia, Germany

**Keywords:** eHealth literacy, digital health literacy, electronic health literacy, electronic health, digital health, geriatric patients, digital inclusion, electronic health information, geriatrics

## Abstract

**Background:**

The rapid growth of digital health technologies has highlighted the importance of eHealth literacy, which is crucial for navigating health information and tools such as telemedicine and eHealth records. However, older adults often have reservations about digital technologies and face barriers due to cognitive, sensory, and motor impairments, which hinder their ability to effectively use digital health applications. Despite its relevance, research on eHealth literacy in older populations is limited, with a particular gap in understanding how self-assessments of eHealth literacy correlate with health characteristics.

**Objective:**

The study seeks to explore eHealth literacy among hospitalized geriatric patients in Germany. The main objective is to evaluate the eHealth literacy level within this particular group and identify the clinical and sociodemographic factors that may influence it.

**Methods:**

Data from this observational study were analyzed to explore characteristics influencing eHealth literacy in hospitalized geriatric patients. The study included patients aged 60 or older in the Department of Geriatric Medicine at Marien Hospital Herne and enrolled in the standardized early rehabilitation pathway. eHealth literacy was measured using the German version of the eHealth Literacy Scale (GR-eHEALS), a self-assessment tool. Sociodemographic and clinical data, including cognitive function (Montreal Cognitive Assessment [MoCA]), frailty (Clinical Frailty Scale [CFS]), and depression (Depression in Old Age Scale [DIA-S]), were collected. Statistical analyses involved descriptive statistics, *t* tests, chi-square tests, and regression analyses to examine the relationship between eHealth literacy and various factors. Sociodemographic and clinical data, including cognitive function (MoCA), frailty (CFS), and depression (DIA-S), were collected.

**Results:**

A total of 244 participants (aged 60‐97, mean 77.2, SD 6.9 years) were included in the analysis. Participants generally reported an eHealth literacy, with higher scores in “information seeking” (mean 3.64, SD 0.84) compared to “information appraisal” (mean 3.32, SD 0.80). Significant differences in eHealth literacy were observed by age, with older participants scoring lower on both subscales. Frailty was significantly associated with critical appraisal of online health information (B=–1.372, 95% CI –2.554 to –0.189), suggesting that individuals who are frail struggle more with evaluating health information. No significant associations were found with other sociodemographic variables, including MoCA scores, education, or gender.

**Conclusions:**

While the findings suggest a positive self-reported ability to engage with digital health tools, the selective sample and reliance on self-assessment limit the accuracy of these results. It also emphasizes the need for tailored interventions to support older adults in improving their eHealth literacy, particularly those with higher levels of frailty. Further research, including longitudinal studies and objective assessments, is essential to better understand and address the digital health needs of the geriatric population.

## Introduction

The use of digital tools is steadily increasing in both everyday life and health care [1,2]. This includes digital health offers like electronic health records, wearable devices to track health-related parameters, telemedicine, and smart hospitals, as well as AI [3]. Digital health, or eHealth, in this context, is defined as the use of modern information and communication technologies to deliver, manage, and support health care services, health information exchange, and clinical documentation across institutional and geographic boundaries. In the context of digital health care and telemedicine, eHealth encompasses a broad range of activities including electronic health records, telemedicine, remote patient monitoring, mobile health, and digital tools for health education, disease prevention, and patient engagement [4,5]. In addition to these primary known developments, there are innovations that so far only affect small areas of daily life, such as deep learning methods, virtual reality and augmented reality, as well as digital informed consent [3].

Since the German Digital Care Act was passed in 2019, digital health applications (DiGA) have been included in the catalog of services covered by statutory health insurance in Germany and can therefore be prescribed by physicians and psychotherapists [6]. Applications that meet the requirements of the Federal Institute for Drugs and Medical Devices (Bundesinstitut für Arzneimittel und Medizinprodukte [BfArM]) with regard to various factors such as safety, quality, and data protection and undergo an appropriate approval process are then intended to support insured persons in the treatment of illnesses and the compensation of impairments [7]. This regulation ensures that DiGA provide users with evidence-based information and support [8]. Health information is increasingly available on the internet, but misinformation is widespread there as well [9]. For example, AI provides evidence-based answers to health questions but also incorrect answers [10] as well as potentially harmful information [11].

Those innovations also raise challenges regarding data privacy and security. In addition, we note that challenges arise when systems are not interoperable, and data flow is not secure. Other organizational issues may include liability and malpractice, as well as regulatory challenges. Personal and psychological barriers are also an obstacle to the use of digitalization [3].

Therefore, as health care services increasingly transition to digital arrangements, individuals are required to develop new skills to use them and participate in health care [12]. Dealing with these challenges requires a wide range of personal skills, which are described under the term eHealth literacy and include analytical competencies (traditional literacy, media literacy, and information literacy) and context-specific competencies (computer literacy, scientific literacy, and health literacy) [13]. eHealth literacy is defined by Norman and Skinner [13] as the ability to seek, find, understand, evaluate, and apply health information from electronic sources to address or solve a health problem. The continuous advancement of digitalization has led to a wide range of additions to the definition by various authors, as well as an update to Norman and Skinner’s definition [14]. These definitions focus primarily on critical perspectives on information and the relevance of individual health literacy, organizational health literacy, and navigational health literacy [15]. These skills are particularly crucial in the context of digital health care and telemedicine, where patients and health care providers alike rely on digital platforms for communication, diagnosis, and treatment options [16]. The importance of eHealth literacy extends beyond just accessing information. For patients, it is essential for self-management of health conditions, particularly in chronic diseases, where patients regularly need to make informed decisions [13,17]. However, studies have shown that these users in particular pose challenges: social and structural determinants of eHealth literacy in digital health care and telemedicine include socioeconomic status (income, education, and employment), age, ethnicity, social support, language proficiency, and access to digital infrastructure (such as broadband internet and devices). Lower income, lower educational attainment, older age, minority ethnicity, and unemployment are consistently associated with lower eHealth literacy scores, which in turn limits effective engagement with telemedicine and digital health tools [18-20]. In addition, lower levels of eHealth literacy may contribute to health disparities and limit the benefits of digital health technologies [19]. Low eHealth literacy is associated with increased barriers to telemedicine adoption, especially among older adults and socially disadvantaged groups, exacerbating health disparities and limiting the reach of digital health innovations [21].

Furthermore, higher levels of eHealth literacy have been associated with better health outcomes, as individuals with greater eHealth literacy are more likely to make informed and accurate health decisions, enhancing their overall well-being [19]. Higher levels of eHealth literacy are additionally associated with improved health-promoting behavior, better self-management, and more effective use of health services [19].

This shows that the level of eHealth literacy is highly relevant, while at the same time its degree of manifestation in society varies greatly. For health care providers, understanding a patient’s level of eHealth literacy is key to delivering effective telemedicine services and ensuring patients can fully engage with digital health interventions [16]. Measurement of eHealth literacy is therefore an area in which a great deal of research is being conducted.

Despite its importance, the evidence on eHealth literacy among the older population remains incomplete, particularly regarding the factors associated with individual differences in eHealth literacy [22]. This group is often at higher risk of difficulties navigating digital health tools [23,24], yet their specific challenges and needs have not been sufficiently addressed. In addition to chronological age, frailty—a multidimensional geriatric syndrome characterized by diminished physiological reserve and increased vulnerability to adverse health outcomes—is increasingly recognized as an important determinant of digital engagement in later life [25]. Frailty frequently coexists with cognitive impairment, functional limitations, and multimorbidity, all of which may compromise an individual’s ability to access, understand, and effectively use digital health technologies [25,26]. However, differences are not solely attributed to age: cognitive, sensory (eg, tactile, visual, or auditory), and motor impairments directly affect one’s ability to interact with and independently use technologies [27]. Cognitive barriers include declines in memory, attention, and information processing speed, as well as mild cognitive impairment and dementia, which can limit comprehension of instructions and the ability to navigate digital platforms, even when cognitive scores are within normal limits [28]. Functional barriers are common and include visual and hearing impairments, reduced manual dexterity (eg, arthritis), and sensorimotor changes, all of which hinder basic interactions with devices such as touchscreens and reading on small screens [27]. Motivational barriers encompass low self-efficacy, resistance to change, generational skepticism, privacy concerns, and a lack of perceived benefit or relevance of digital health tools. Many older adults report frustration, confusion, and mistrust in digital platforms, which can demotivate engagement [29,30]. Additional factors such as limited digital literacy, lack of awareness, complicated application interfaces, and insufficient support networks further exacerbate these barriers [31].

Nevertheless, people with cognitive impairments also use digital devices [32]. They expect time savings, report convenience and faster access to care as perceived advantages of digital health solutions [14]. However, in order to meet these positive expectations, usage must be successful, which may be difficult due to the obstacles mentioned above. Building on the recognition that digital health solutions can offer significant benefits, it is crucial to explore how these solutions can be used by those who face cognitive challenges, such as hospitalized geriatric patients.

This study aims to address this gap by examining eHealth literacy in a sample of hospitalized geriatric patients in Germany. The primary goal is to assess the level of eHealth literacy in this specific population and identify the clinical and sociodemographic characteristics that influence it.

Hypotheses of the study were:

- Self-reported eHealth literacy does vary by age.
- Participants with lower functional status and higher frailty report lower levels of eHealth literacy scores.

## Methods

### Study Design and Setting

The reporting of this observational study followed the Strengthening the Reporting of Observational Studies in Epidemiology (STROBE) guidelines [33]. The presented data are from an observational study of the DiGA4Aged project (dDiGA for older people with depressive disorders). The project consists of a pilot study [34], an observational study, and a randomized controlled trial (RCT) [35]. It aims to examine the feasibility, acceptance, and clinical relevance of a DiGA for older adults with depressive disorders.

The quantitative, observational study was designed to explore clinical and sociodemographic factors associated with digital media use and DiGA engagement among hospitalized geriatric patients. For this analysis, only data regarding eHealth literacy were used. Data collection took place in the Department of Geriatric Medicine at Marien Hospital Herne, Germany, and included both inpatient care and the geriatric day clinic. Throughout the study period (January to December 2024), all consecutively admitted patients were assessed for eligibility and invited to participate. Detailed methodological information on this screening study as well as the preceding pilot study is available in a previously published joint study protocol [36]. There have been no deviations from the planned and reported procedure.

### Patients’ Characteristics and Recruitment

Participants qualified for inclusion if they were at least 60 years old, admitted to the Department of Geriatric Medicine at Marien Hospital Herne in Germany, enrolled in the standardized geriatric early rehabilitation pathway (rehabilitation during acute care), which requires a minimum hospital stay of 14 days, and able to provide written informed consent. Patients were included if they reported using the internet to search for health information. Exclusion criteria included an anticipated hospital stay shorter than 14 days, insufficient cognitive ability to give informed consent, or refusal to participate. There were no restrictions concerning gender or specific medical diagnoses.

Recruitment was conducted consecutively throughout the study period of the entire year 2024. Eligible patients were identified through the hospital information system and personally contacted by trained research staff within the last three days before their planned discharge.

Study participation involved a 2-step process: first, the use of routinely collected clinical data, and second, the collection of additional information through standardized questionnaires for patients consenting to the extended data acquisition. For the reported analyses, only patients who completed both steps and the questionnaire regarding eHealth literacy were included.

### Measurements

Trained study staff visited all patients with a tablet computer and conducted the survey in a structured interview format. Participants provided their responses verbally, and the study staff entered the answers directly into the electronic data capture system. The Montreal Cognitive Assessment (MoCA) was administered separately using the standard paper-based format, as some tasks (eg, drawing) require written responses. eHealth literacy was measured using the German version of the eHealth Literacy Scale (GR-eHEALS) [37], the revised GR-eHEALS developed by Norman and Skinner [13]. The GR-eHEALS was first developed including 16 items, with the first 8 items corresponding to the original eHEALS, assessing individuals’ ability to find, evaluate, and apply online health information. Items 9‐16 were based on the eHEALS-E questionnaire from Petrič et al [38]. However, validation was only successful for items 1‐8.

Responses were recorded on a 5-point Likert scale ranging from “strongly disagree” (1) to “strongly agree” (5). The scale includes 2 validated subscales: the Information Seeking scale (items 1‐4) and the Information Appraisal scale (items 5‐8). Both scales were scored by summing the respective item responses to generate a total score for each subscale [37]. This paper reports only data from the original eHEALS (items 1‐8). The information seeking scale achieved a satisfactory Cronbach α of 0.92, and the information appraisal scale achieved a satisfactory Cronbach α of 0.92 and 0.83 [37].

The GR-eHEALS was chosen since it is a validated instrument to measure eHealth literacy. However, due to the complexity of the content dimensions, the operationalization of eHealth literacy is conceptually challenging. A systematic review by Xie et al [39] also highlighted the widespread use of eHEALS as a primary assessment tool for eHealth literacy. Moreover, a review by Huang et al [40] discussed various tools for measuring eHealth literacy in older adults, including eHEALS. Their findings suggest that while these tools are valuable, there is a need to strengthen their implementation in clinical practice. Based on the positive assessment by Huang et al [40] among the existing options, eHEALS was selected for this study. While self-assessment is a valuable tool for understanding an individual’s perceived competency, it has inherent limitations. Subjective measures often overestimate actual eHealth literacy, as individuals may report high confidence without possessing the necessary skills to effectively navigate, evaluate, or apply online health information in real-world scenarios [41,42].

Furthermore, sociodemographic data from the geriatric assessment conducted routinely within the first 48 hours of admission: age, sex, years of education (self-reported), MoCA (to assess cognitive function) [43], Barthel Index (to assess functional status) [44], Clinical Frailty Scale (CFS) [45,46], living situation prior to admission (alone vs with others), Depression in Old Age Scale (DIA-S, to assess depression symptoms) [47], and care degree on admission. MoCA, Barthel Index, and DIA-S were assessed both at admission and at discharge. For this analysis, discharge values were used whenever available, as they were considered to better reflect the patients’ stable cognitive, functional, and psychological status following recovery from the acute illness. If a discharge assessment was unavailable (eg, because the participant declined retesting), the corresponding admission value was used. Frailty was assessed using the CFS, a 9-point clinical scale ranging from 1 (nonfrail) to 9 (severely frail), with higher scores indicating greater levels of frailty and vulnerability [45,46]. For the purpose of statistical analyses, the CFS was dichotomized into nonfrail (CFS 1‐4) and frail (CFS 5‐9). The assessment was performed as part of routine geriatric clinical care by trained geriatric physicians and study nurses according to standardized procedures within the department.

The care degree is part of the German long-term care insurance system that regulates reimbursement for care and reflects the individual’s level of care dependency based on impairments in independence across 6 domains of daily functioning. Care degrees range from 1 (minor impairment of independence) to 5 (most severe impairment requiring extensive care).

### Ethical Considerations

The DiGA4Aged project received ethical approval from the Medical Faculty of Ruhr-University Bochum on November 17, 2023 (registration number 23‐7901). The observational study was conducted according to the Declaration of Helsinki. Data were recorded and managed using the web-based electronic data capture system REDCap (Vanderbilt University) [48].

### Data Handling and Statistical Analysis

No separate a priori sample size calculation or formal power analysis was conducted. The analyzed cohort originated from the DiGA4Aged screening study, which was primarily designed to identify and recruit eligible participants for the subsequent pilot study and RCT. Consequently, the duration and target sample size of the screening study were prospectively determined by the expected number of screened geriatric patients required to achieve the planned recruitment for the intervention study, as described in the published study protocol [36]. In accordance, all eligible geriatric patients admitted during the predefined one-year recruitment period were consecutively invited to participate. The current analyses represent an exploratory analysis of this screening cohort. All questions were administered in person during the participants’ hospital stay. Statistical analyses were performed with SPSS statistical software (IBM Corp, SPSS Statistics for Windows, version 29.0).

Descriptive statistics were used to characterize the study sample. Continuous variables were summarized using medians, means, and SD. Categorical variables were presented as absolute and relative frequencies (n, %). To assess whether the distribution of values in the subsample differed from the distribution observed in the overall study population, different one-sample procedures were applied depending on the scale level of the variables. For metric variables, one-sample *t* tests were conducted to determine whether the mean of the subsample significantly deviated from the mean of the total sample. For categorical variables with exactly 2 response categories, one-sample binomial tests were performed. These tests evaluated whether the observed proportions in the subsample differed from those in the overall sample. For categorical variables with more than 2 categories, one-sample chi-square goodness-of-fit tests were used. In these analyses, the observed category frequencies of the subsample were compared with the expected frequencies derived from the distribution of the full sample. This approach allowed for evaluating deviations across the entire categorical distribution rather than testing individual categories separately.

Item-level statistics for the GR-eHEALS were calculated, including medians, means, SDs, and the distribution of response categories. To test for group differences, *t* tests and ANOVA were conducted. Participants were categorized based on predefined cut-off values or the median, shown in Table 1 and Multimedia Appendix 1. For variables with established clinical thresholds, participants were divided into 2 groups according to predefined cut-off values: cognitive impairment was defined as a MoCA score <26 [43], frailty as a CFS score ≥5 [45,46], and clinically relevant depressive symptoms as a DIA-S score ≥3 [47]. For continuous variables without established cut-offs, such as age, years of education, and Barthel Index, participants were split using the median value. Sex was categorized as male and female, and living situation was grouped into living alone and living with others. Subsequently, linear regression analyses were performed to examine associations between eHealth literacy and various participant characteristics. Prior to the regression analyses, multicollinearity was assessed using variance inflation factors (VIF). In addition, residual plots were visually inspected to evaluate linearity, homoscedasticity, and normality of residuals. No major violations of the regression assumptions were identified. Frailty was entered into the regression models as a dichotomous variable based on the CFS (nonfrail: CFS 1‐4 vs frail: CFS 5‐9). These analyses aimed to explore how sociodemographic and clinical factors relate to eHealth literacy levels in the study population. For the remaining categorical variables, the same cut-off values or medians described above were applied. Continuous variables were included in the regressions in their original scale. As the analyses were exploratory in nature, no correction for multiple testing was applied. Therefore, all statistical tests should be interpreted as exploratory and hypothesis-generating. The overall model fit was assessed using *R*² and adjusted *R*² values.

**Table 1.** Categorization to test for group differences, *t* tests and ANOVA.

Variables	Categories
Variables with established clinical thresholds	
Montreal Cognitive Assessment.	1‐25 (indication of cognitive impairment) 26‐30 (no indication of cognitive impairment)
Clinical Frailty Scale	1‐4 (nonfrail to mildly frail) 5‐9 (moderately to severely frail)
Depression in Old Age Scale	0‐2 (no or minimal depressive symptoms) 3‐10 (elevated depressive symptoms)
Continuous variables divided according to median value	
Age	≤77 years >77 years
Years of education	≤11 years >11 years
Barthel Index	≤75 >75
Categorical variables	
Sex	Male Female
Living situation	Living alone Living with others

## Results

### Characteristics of Study Participants

Throughout the study period, all patients fulfilling the inclusion criteria were consecutively invited to participate. The following procedure was used to identify patients eligible for inclusion: 1499 individuals were approached for the study. A total of 1134 of them gave written informed consent for the use of routinely collected clinical data for research purposes. Among these, 795 patients additionally agreed to take part in the supplementary study-related data collection. Of these 795 patients, 255 reported using the internet for health-related information and therefore answered the GR-eHEALS. This group of patients forms the study population of the reported substudy described below.

After excluding cases with incomplete responses, a total of 244 fully completed GR-eHEALS questionnaires were included in the final analyses, as illustrated in Figure 1. The characteristics of the study population for this substudy are summarized in Table 2. In total, participants’ age ranged from 60 to 97 years (mean age 77.2, SD 6.9 years), with females accounting for 121 out of 244 (49.6%) of the sample. To show the relation between the subgroup of 244 patients and the entire cohort, we provide the characteristics in Table 2. In the following, we compare the subgroup to the group of nonsurfers to point out the differences.

**Figure 1.**
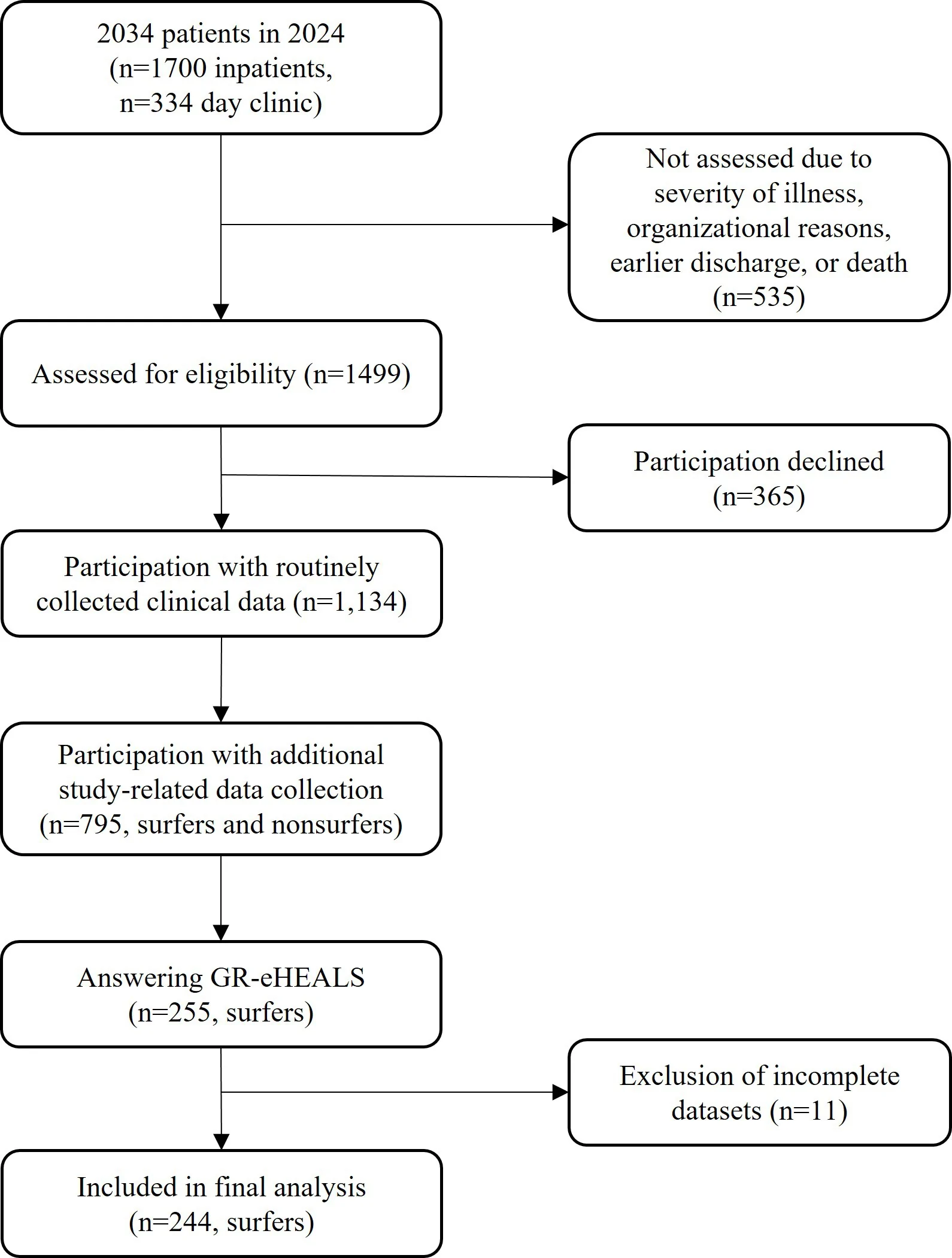
Consolidated Standards of Reporting Trials diagram. GR-eHEALS: German version of the eHealth Literacy Scale.

**Table 2.** Characteristics of the entire cohort, study participants, and group comparisons.

Characteristics	Entire cohort (n=1134)	GR-eHEALS-sample (N=244)	*P* value^a^
Age, mean (SD) years	79.2 (14.8)	77.2 (6.9)	.54
Sex, n (%)			
Male	398 (35.1)	123 (50.4)	<.001
Female	736 (64.9)	121 (49.6)	.32
Years of education (self-reported), mean (SD)	10.8 (2.3)	11.9 (2.4)	.047
MoCA^b^, mean (SD)	17.2 (6.2)	19.7 (6.8)	.002
Barthel Index^c^, mean (SD)	69.7 (18.4)	73.3 (18.7)	<.001
Clinical Frailty Scale, mean (SD)	5.6 (1.0)	5.4 (1.1)	.13
DIA-S^d^, mean (SD)	3.2 (2.6)	2.3 (2.5)	.47
Care degree on admission, n (%)			<.001
None	322 (28.4)	88 (36.1)	
1	122 (10.8)	26 (10.7)	
2	441 (38.9)	85 (34.8)	
3	224 (19.8)	40 (16.4)	
4	19 (1.7)	3 (1.2)	
5	2 (0.2)	0	
Living situation prior to admission^e^, n (%)			.006
Alone	452 (56.9)	122 (50.0)	
With others (in private household)	318 (40.1)	119 (48.8)	
Nursing home	24 (3.0)	3 (1.2)	
Setting of recruitment, n (%)			
Inpatient care	958 (84.5)	198 (81.1)	.04
Geriatric day clinic	176 (15.5)	46 (18.9)	<.001

a Comparison of the study participants (n=244) and the population in the overall study (nonsurfers, n=890).

b MoCA: Montreal Cognitive Assessment.

c Barthel Index 0-100, with 100 indicating complete independence and 0 indicating no independence.

d DIA-S: Depression in Old Age Scale.

e Missing values in up to 64 patients were only recorded in a subsample of 794 participants due to methodological reasons

### Comparison of the Substudy Participants and the Population in the Overall Study

To interpret the results in relation to the overall DiGA4Aged study and the total geriatric population, we compared the study participants reported here with the overall screening cohort of the substudy (n=1134). The overall screening cohort included both the study participants and patients who were excluded from this substudy because they did not use the internet to search for health-related information (referred to as “nonsurfers”). Although these nonsurfers did not complete the GR-eHEALS, they participated in the screening study by providing consent for the use of their routinely collected clinical data, allowing for the characterization of the study sample within the overall screening cohort. In the following and for descriptive purposes, we refer to the participants included in the substudy as “study participants” and to the nonparticipants as “nonsurfers” for simplicity. Nonsurfers are the 1134 people who participated in the routine data collection, minus the 244 people who completed the eHEALS questionnaire. This means that 890 nonsurfers are compared with 244 study participants.

When comparing the study participants with the overall screening cohort, the study participants were younger and more often male than the nonsurfers. The study participants showed better cognitive and functional status compared to the nonsurfers. Specifically, the study participants had significantly higher MoCA scores (19.7 vs 17.2; *P*=.002) and a higher Barthel Index (73.3 vs 69.7; *P*<.001) than the nonsurfers, suggesting greater independence in activities of daily living. A notable difference was observed in the distribution of care degrees on admission, with the study participants having fewer participants with a care degree of 3 or higher (*P*<.001) than the nonsurfers. Finally, the sample of study participants also showed significant differences in living arrangements prior to admission, with more participants living with others or in a private household, and fewer living alone compared to the nonsurfers (*P*=.006). Details are provided in Table 2.

### eHEALS Items and Scales

When examining eHealth literacy, the responses from participants on the GR-eHEALS offer a nuanced picture of their abilities to find, evaluate, and apply online health information. Looking at the individual items, participants report a broad range of self-perceived eHealth literacy, with responses spanning the full range of the 5-point Likert scale from “strongly disagree” (1) to “strongly agree” (5), shown in Table 3. For example, item 2, “I know how to use the internet to find answers to my health questions,” received a high level of agreement, with a mean score of 3.79 (SD 0.97). However, some items, such as item 8, “I feel confident using internet information to make health decisions,” rated lower and were more varied, with a broader spread of responses, especially leaning towards neutrality or disagreement, as indicated by the mean score of 2.85 (SD 1.12). The mean scores for individual items ranged up to 3.79 (item 2: “I know how to use the internet to find answers to my health questions”), with SDs varying from 0.97 to 1.17, indicating some variability in responses. The overall distribution of answers showed that most participants tended to agree or strongly agree with the statements about their eHealth literacy, with items 1‐4 (subscale Information Seeking) generally receiving higher agreement rates than items 5‐8 (subscale Information Appraisal), which assessed critical appraisal of health information.

**Table 3.** Overview of item and scale statistics (N=244)^a^.

Original item in German (English translation)	Median(IQR)	Mean (SD)	Response distribution, n (%)					Sum (mean [SD])/max
			1	2	3	4	5	
Information seeking								
Ich weiß, wie ich Internetseiten mit hilfreichen Gesundheitsinformationen finden kann. (I know how to find websites with helpful health information.)	4 (4-4)	3.75 (0.98)	14 (5.7)	19 (7.8)	13 (5.3)	165 (67.6)	33 (13.5)	
Ich weiß, wie ich das Internet nutzen kann, um Antworten auf meine Gesundheitsfragen zu erhalten. (I know how to use the internet to find answers to my health questions.)	4 (4-4)	3.79 (0.97)	11 (4.5)	23 (9.4)	11 (4.5)	160 (65.6)	39 (16.0)	
Ich weiß, welche Seiten mit Gesundheitsinformationen im Internet verfügbar sind. (I know which websites with health information are available on the Internet.)	4 (2-4)	3.27 (1.17)	25 (10.2)	48 (19.7)	26 (10.7)	126 (51.6)	19 (7.8)	
Ich weiß, wo ich im Internet hilfreiche Gesundheits-informationen finden kann. (I know where to find helpful health information on the Internet.)	4 (4-4)	3.73 (1.01)	10 (4.1)	29 (11.9)	19 (7.8)	145 (59.4)	41 (16.8)	
Scale: information seeking	4 (3.25-4)	3.64 (0.84)	15 (6.1)	30 (12.3)	17 (7.0)	149 (61.1)	33 (13.5)	14.5 (3.4)/20
Information appraisal								
Ich weiß Gesundheitsinformationen aus dem Internet so zu nutzen, dass sie mir weiterhelfen. (I know how to use health information from the internet in a way that helps me.)	4 (3-4)	3.46 (1.07)	11 (4.5)	45 (18.4)	39 (16.0)	119 (48.8)	30 (12.3)	
Ich bin in der Lage, Internetseiten mit Gesundheitsinformationen kritisch zu bewerten. (I am able to critically evaluate websites with health information.)	4 (3-4)	3.60 (1.01)	12 (4.9)	29 (11.9)	34 (13.9)	139 (57.0)	30 (12.3)	
Ich kann zwischen vertrauenswürdigen und fragwürdigen Internetseiten mit Gesundheitsinformationen unterscheiden. (I can distinguish between trustworthy and questionable websites with health information.)	4 (2-4)	3.39 (1.09)	14 (5.7)	49 (20.1)	32 (13.1)	125 (51.2)	24 (9.8)	
Ich fühle mich sicher darin, Informationen aus dem Internet zu nutzen, um Entscheidungen in Bezug auf meine Gesundheit zu treffen. (I feel confident using information from the internet to make decisions about my health.)	3 (2-4)	2.85 (1.12)	27 (11.1)	82 (33.6)	46 (18.9)	79 (32.4)	10 (4.1)	
Scale: information appraisal	3.5 (3-4)	3.32 (0.80)	16 (6.6)	51 (20.9)	38 (15.6)	115 (47.1)	24 (9.8)	13.3 (3.2)/20
Scale: GR-eHealth Literacy (item 1‐8)	3.6 (3.25-4)	3.48 (0.76)	16 (6.6)	40 (16.4)	28 (11.4)	132 (54.1)	28 (11.5)	27.8 (6.1)/40

a Response distribution: 1=strongly disagree, 2=disagree, 3=neutral, 4=agree, 5=strongly agree.

To calculate the overall eHealth literacy score, the summed scores of Items 1‐8 (Information Seeking and Information Appraisal) were used, with a possible range from 8 to 40 points and 4 to 20 points for the subscores, respectively, divided by the number of items. The mean score for the eHealth literacy scale was 3.48 (SD 0.76); for the Information Seeking subscale (Items 1‐4), it was 3.64 (SD 0.84), and for the Information Appraisal subscale (Items 5‐8), it was slightly lower (mean 3.32, SD 0.80). In Table 3, absolute frequencies of sum-scores were rounded to whole persons. Due to this rounding, minimal discrepancies (+1 person, 2%) may occur. To maintain a consistent sample size of n=244 (100%), the response option with the largest proportion was reduced by one person (2%; following standard rounding rules).

To examine differences in eHealth literacy across the subscales, independent *t* tests were conducted for each scale, using predefined cut-off or median values. Significant differences were only found for age across all scales, with *t*_242_=2.365; *P*=.02 for the Information Seeking scale; *t*_242_=2.544; *P*=.012 for the Information Appraisal scale; and *t*_242_=2.652; *P*=.009 for the whole eHealth Literacy scale. In addition, frailty was significantly associated with the Information Appraisal scale, where participants with higher frailty scores (frailty 5‐9) demonstrated lower scores on the Information Appraisal subscale compared to those with lower frailty scores (frailty 1‐4), with *t*_242_=2.067; *P*=.040. For all other comparisons, including MoCA, DIA-S, years of education, Barthel Index, gender, care degree, and living situation, no significant differences were found across the scales.

### Characteristics Influence on eHealth Literacy

To investigate the influence of various characteristics on eHealth literacy, linear regression analyses were conducted for all scales (Information Seeking, Information Appraisal, and the overall eHealth Literacy), see Table 4. Due to missing data, regression analyses were based on complete cases, resulting in 207 participants included in the regression models. For the Information Seeking scale, the regression model explained 3.2% of the variance (*R*²=0.032, adjusted *R*²=–0.012). No individual predictor showed a significant association with information seeking. For the Information Appraisal scale, the model explained 6.6% of the variance (*R*²=0.066, adjusted *R*²=0.024). Frailty emerged as significantly negatively associated with Information Appraisal (B=–1.372; *P*=.02), indicating that individuals with higher frailty scores were less likely to critically appraise online health information. However, no other characteristics showed significant association with the participants’ ability to assess health information. The regression model for the overall eHealth literacy score (Items 1‐8) explained 5.3% of the variance (*R*²=0.053, adjusted *R*²=0.010). Frailty showed a marginally significant negative association (B=–2.217; *P*=.05).

**Table 4.** Linear regression analysis of factors associated with eHealth literacy^a^.

Characteristics	Sum score for eHealth literacy (GR-eHEALS)^b^			
	B^c^	SE	Standardized β (95% CI)	*P* value
Scale information seeking (item 1‐4)^d^				
Age	−0.040	0.036	−0.082 (−0.111 to 0.031)	.27
Sex (male)	−0.084	0.512	−0.012 (−1.094 to 0.926)	.87
Years of education	0.067	0.106	0.047 (−0.142 to 0.276)	.53
MoCA^e^	0.069	0.668	0.007 (−1.249 to 1.388)	.92
Barthel Index	0.009	0.014	0.050 (−0.019 to 0.038)	.51
Frailty Score	−0.845	0.635	−0.097 (−2.097 to 0.407)	.19
DIA-S^f^	−0.059	0.518	−0.008 (−1.081 to 0.963)	.91
Care degree on admission	−0.143	0.224	−0.049 (−0.585 to 0.299)	.53
Living situation (alone)	−0.012	0.506	−0.002 (−1.011 to 0.986)	.98
Scale information appraisal (item 5‐8)^g^				
Age	−0.049	0.034	−0.105 (−0.116 to 0.018)	.15
Sex (male)	−0.630	0.484	−0.096 (−1.583 to 0.324)	.19
Years of education	0.045	0.100	0.033 (−0.153 to 0.242)	.65
MoCA	0.046	0.631	0.005 (−1.199 to 1.291)	.94
Barthel Index	0.018	0.013	0.099 (−0.009 to 0.044)	.18
Frailty Score	−1.372	0.600	−0.163 (−2.554 to −0.189)	.02
DIA-S	−0.068	0.490	−0.010 (−1.034 to 0.897)	.89
Care degree on admission	−0.038	0.212	−0.013 (−0.455 to 0.380)	.86
Living situation (alone)	−0.296	0.478	−0.045 (−1.239 to 0.647)	.54
Scale eHealth Literacy (item 1‐8)^h^				
Age	−0.088	0.065	−0.101 (−0.216 to 0.039)	.17
Sex (male)	−0.713	0.920	−0.058 (−2.529 to 1.102)	.44
Years of education	0.111	0.190	0.043 (-0.264 to 0.487)	.56
MoCA	0.115	1.201	0.007 (−2.254 to 2.484)	.92
Barthel Index	0.027	0.026	0.080 (−0.023 to 0.078)	.29
Frailty score	−2.217	1.141	−0.140 (−4.467 to 0.034)	.05
DIA-S	−0.127	0.932	−0.010 (−1.965 to 1.710)	.89
Care degree on admission	−0.181	0.403	−0.034 (−0.975 to 0.614)	.66
Living situation (alone)	−0.308	0.910	−0.025 (−2.102 to 1.486)	.74

a From 1.077 to 1.191, indicating no relevant multicollinearity.

b GR-eHEALS: German version of the eHealth Literacy Scale.

c B: unstandardized regression coefficient.

d Scale information seeking (items 1-4): *R*2=0.032, adjusted *R*2=–0.012, *F*_9,197 _=0.729; *P*=.68.

e MoCA: Montreal Cognitive Assessment.

f DIA-S: Depression in Old Age Scale.

g Scale information appraisal (items 5-8): *R*2=0.066, adjusted R*R*2 = 0.024, *F*_9,197_=1.551; *P*=.13.

h Scale eHealth Literacy (items 1-8): *R*2=0.053, adjusted *R*2=0.010, *F*_9,197_=1.226; *P*=.28.

## Discussion

### Main Findings

The study aimed to explore eHealth literacy among geriatric patients, focusing on how sociodemographic and clinical factors may influence it. Patients included were 60‐97 years old and reported using the internet to search for health-related information. The findings suggest that older adults, particularly those with higher levels of frailty, tend to have lower eHealth literacy scores, particularly in the area of information appraisal. It partially supports our hypothesis, as frailty was significantly associated with lower information appraisal scores in the multivariate analysis. However, no significant gender differences in eHealth literacy were observed, which suggests that digital socialization or gender stereotypes may not be as impactful in this cohort. Furthermore, despite expectations, age was not significantly associated with eHealth literacy in this study, which may reflect the stability of self-assessments despite fluctuating objective digital skills. Although age was significantly associated with eHealth literacy in the bivariate analyses, this association was no longer significant in the multivariate regression models. This suggests that chronological age alone cannot independently explain the differences in eHealth literacy. In our geriatric sample, frailty was significantly associated with the ability to critically evaluate online health information, suggesting that functional health status may be more relevant than chronological age. Given the close association between age and frailty, the bivariate association with age may partly reflect age-related differences in functional status rather than an independent effect of age itself. Although frailty was significantly associated with the information appraisal dimension of eHealth literacy, the regression models explained only a limited proportion of variance. This suggests that additional factors beyond the sociodemographic and clinical characteristics examined may contribute to differences in eHealth literacy among geriatric patients. Potential factors that were not assessed in this study include previous experience with digital technologies, digital skills, general health literacy, social support and attitudes, and motivation towards digital technologies. However, only 255 of 795 participants (32%) in our study used the internet to gather health-related information, demonstrating the age gap in this area. These findings indicate that a considerable proportion of hospitalized geriatric patients do not use the internet as a source of health information and may therefore not currently benefit from digital health services. Several studies have examined eHealth literacy in older populations, with varying methodologies and findings. For example, Jørgensen et al (2023) [49] reported that many hospitalized geriatric patients had limited computer habits and digital literacy, with only half regularly using computers and many unfamiliar with basic digital functions. They also identified that higher digital literacy was associated with less computer-related stress and more frequent use of the internet, particularly for health-related tasks. This aligns with our findings, which showed that participants with lower levels of frailty demonstrated higher eHealth literacy scores, especially in the area of information appraisal. However, our study extends Jørgensen et al’s [49] work by emphasizing the specific challenges faced by individuals who are frail in critically appraising online health information, which was a key finding in our analysis. While their study primarily focused on general digital skills, our results highlight that eHealth literacy may not only be related to basic computer skills but may also be associated with physical and cognitive health factors. In particular, frailty was linked to lower confidence in critically appraising online health information.

Arcury et al [50] showed that higher levels of eHealth literacy, measured by eHEALS, were associated with better health knowledge and more frequent use of health information sources. It is plausible that participants who used digital health tools more frequently may have had higher eHealth literacy scores, although we cannot directly confirm this in our data.

Self-assessment may have introduced a self-report bias. A major gap in the current research is the predominance of subjective self-assessment tools, with relatively few studies using objective, performance-based measures of eHealth literacy. This limits the ability to accurately predict actual usage behavior and identify those at risk for poor engagement or adverse outcomes in digital health care and telemedicine [32-34]. Therefore, understanding the role of subjective self-assessments in the broader picture of eHealth literacy is crucial. A review by Azizan [29] showed that most of the available tools (57%) currently involve self-assessment. Such tools often require significantly less time and organizational effort, as well as evaluation time, than a performance test, which would enable more objective results. Therefore, it represents a tool that can be realistically used in practice. Since no validated German instrument for objective evaluation exists, this approach was chosen. It offers first insights into the needs of the geriatric population, since the self-assessment can show the need for support, either knowledge improvement or empowerment to enable safe digital health engagement.

With regard to self-assessment, an effect that has been described extensively in the literature may come into play: the Dunning-Kruger effect, which is characterized by 2 mechanisms: First, a lack of knowledge means that people are unaware of their own gaps in knowledge, causing them to overestimate their abilities. Second, greater competence increases awareness of how much one does not yet know, resulting in a more cautious self-assessment [51]. This phenomenon is particularly relevant in the context of eHealth literacy. Older adults may feel confident in their ability to use digital tools, even when their skills do not align with their perceptions. For instance, a person might believe they are proficient at navigating health apps or searching for medical information online, but in reality, they may struggle with critical aspects like evaluating the credibility of sources or understanding complex health data. This misalignment between perceived and actual competence often arises from the first mechanism of the Dunning-Kruger effect, lack of awareness about one’s own limitations. The phenomenon suggests that subjective self-assessments might not always accurately reflect actual eHealth literacy, particularly in individuals who are frail, in whom functional limitations may affect confidence and perceived abilities to engage with digital health information. Interestingly, although no significant gender differences were found, this may be due to the complexity of digital socialization and the fact that both men and women in our sample may have had similar levels of exposure to digital health tools. The lack of significant differences in age and self-reported eHealth literacy could be attributed to the stability of self-perception despite fluctuating digital abilities. This suggests that subjective self-assessments may not always accurately reflect actual eHealth literacy, particularly in individuals who are frail, whose functional status and cognitive abilities are compromised. For a more objective measurement, individuals could first be asked to assess their own digital literacy, which could then be verified by testing their actual abilities. By comparing self-assessments with real-world performance, a clearer understanding of their true eHealth literacy can be achieved.

Our results can also be supported in part by the use of a framework. Richardson et al (2022) [52] have developed the “Framework for digital health equity,” which offers a systematic approach to making digital interventions more equitable by taking social, structural, and technological aspects into account. The framework takes into account cumulative and interactive effects across multiple domains, as well as risk and resilience factors. Among other factors, the framework states that socioeconomic status, functional status, and access to support are crucial for the successful use of digital offerings for health-related purposes. This cannot be seen in our data since there were no significant associations found with living situation. From the authors’ perspective, the idea that having a reliable support system—whether family members or caregivers—can facilitate better engagement with digital health tools is, however, very convincing. In this context, Hunsaker et al [53] investigated the types of support older adults receive when using technology. They identified informal support (eg, spouses, adult children, grandchildren, and siblings) and formal support (eg, computer classes, technical support staff in stores and via remote access). However, the study also noted that a major problem in this regard is that support is often not provided immediately. This may explain the results, as there are a variety of support options available that are not exclusively tailored to the individual’s living situation. This is certainly an exciting area to be further explored.

The relevance of frailty is also reflected in this framework. However, we found that frailty was significantly associated with the information appraisal dimension of eHealth literacy, an aspect that has received less attention in previous studies. One possible explanation is that the interaction between cognitive and physical limitations may reduce confidence and perceived ability to effectively navigate and critically evaluate online health information.

### Outlook and Requirements

Our findings support the applicability of a validated instrument for assessing eHealth literacy in geriatric populations, reinforcing its utility for measuring digital literacy in older adults despite its limitations as a self-assessment instrument. The variability in scores across studies indicates that subjective assessments may not fully capture the complexities of digital engagement, especially when factors like frailty and cognitive function are involved. Future research should aim to combine subjective self-assessments with more objective, performance-based measures of eHealth literacy such as task-based evaluations, simulated digital health scenarios, or observed information appraisal tasks. Longitudinal studies that track changes in digital literacy over time and their impact on health outcomes would provide valuable insights into how eHealth literacy evolves with age and frailty. Future studies should investigate factors such as digital skills and general health literacy in addition to sociodemographic and clinical characteristics to develop a more comprehensive understanding of the determinants of eHealth literacy among geriatric patients. In view of the rise of AI and the increase in misinformation on the internet, as well as the difficulty of distinguishing it from true information, the skills required are also changing, merging into the meta-competence of eHealth literacy. This is also evident in the ongoing development of the revised eHEALS [14]. This digital progress will require continuous adjustments to education and operationalization.

Further exploration is also needed to evaluate the effectiveness of interventions designed to improve eHealth literacy in older adults, as they require human support, especially those with better eHealth literacy eg, as part of nursing. It would be beneficial to assess how these interventions are implemented and whether they address the specific barriers faced by older adults in digital health settings. Previously, searching the internet for health information required typing, reading, and appraisal of such information. Current developments in the area of AI are already changing these requirements fundamentally and may contribute to digital health equity in the future. However, it will be interesting to observe how the already widespread use of AI in the younger population will change the extent and depth of health-associated applications in the older population.

### Strengths and Limitations

This study has several strengths, including the use of a validated instrument for assessing eHealth literacy (GR-eHEALS). Although the validity of self-measurement is limited in this case, the gold standard in the German validated version was used for self-reported digital health literacy. In addition, the study’s large cohort of 244 participants strengthens the robustness of the findings within the context of geriatric patients who engage with digital health tools. The sample also includes a broad age range (60‐97 years), which allows for the investigation of potential variability in eHealth literacy across different age groups. Despite the self-reported nature of the data, the use of the GR-eHEALS allowed for a standardized approach to assess key aspects of eHealth literacy, including information seeking and critical appraisal.

However, there are weaknesses that must be considered when interpreting the results. The reliance on self-assessment as the sole measure of eHealth literacy is a significant limitation. Self-reported data can lead to overestimations of actual eHealth literacy, as participants may perceive themselves as more capable than they are in practice [41,42]. The absence of objective measures to validate these self-assessments limits the accuracy of the findings. In addition, these analyses were restricted to participants who reported using the internet to obtain health-related information, as completion of the GR-eHEALS requires prior engagement with digital health resources. Consequently, the study population represents a selected subgroup of digitally engaged geriatric patients rather than the entire geriatric inpatient population. This selection may have resulted in higher average eHealth literacy scores than would be expected in unselected hospitalized older adults and limits the generalizability of the findings to patients with little or no experience using digital health technologies. Furthermore, while the study included a relatively large sample, the population is not fully representative of the broader geriatric hospitalized patient group. The participants in this study were notably fitter than the average geriatric patient, as evidenced by their higher scores on cognitive and functional assessments. Therefore, the results must be interpreted with caution, as they may not reflect the experiences of the wider geriatric population, especially those with greater frailty or cognitive impairment. No adjustment for multiple comparisons was performed. Consequently, statistically significant findings should be interpreted with caution and require confirmation in future hypothesis-driven studies.

### Conclusion

This study provides important insights into eHealth literacy in geriatric patients and its associated factors and underscores the importance of interventions that consider the unique challenges faced by older adults, particularly those with frailty. Future research should focus on integrating subjective and objective measures of eHealth literacy, exploring the impact of social support, and evaluating the effectiveness of current interventions aimed at enhancing digital health engagement among older populations.

## Supplementary material

10.2196/92045 Checklist 1 Strengthening the Reporting of Observational Studies in Epidemiology (STROBE) checklist.

## Abbreviations

BfArM: Federal Institute for Drugs and Medical Devices
CFS: Clinical Frailty Scale
DIA-S: Depression in Old Age Scale
DiGA: Digital health application
eHEALS-R: revised eHealth Literacy Scale
GR-eHEALS: German version of the eHealth Literacy Scale
MoCA: Montreal Cognitive Assessment
STROBE: Strengthening the Reporting of Observational Studies in Epidemiology guideline
VIF: variance inflation factors

## References

[1] Recommendations on digital interventions for health system strengthening. World Health Organization.

[2] Theobald P, Herold F, Gronwald T, Müller NG (2026). Use of digital health technologies in Germany [Article in German]. Präv Gesundheitsf.

[3] Shapoo N, Shapoo N, Rehman A, Boma N (2025). Smart healthcare: the role of digital health in modern medicine. Health Care Sci.

[4] Knaup P, Benning NH, Seitz MW, Eisenmann U (2020). eHealth and clinical documentation systems. Stud Health Technol Inform.

[5] Pagliari C, Sloan D, Gregor P (2005). What is eHealth (4): a scoping exercise to map the field. J Med Internet Res.

[6] Digital health applications (DiGA) [Webpage in German]. Federal Ministry of Health.

[7] DiGA und DiPA [Wepage in German]. Federal Institute for Drugs and Medical Devices.

[8] DiGA guidelines (as of December 10, 2025, version 3.6) [Webpage in German]. Federal Institute for Drugs and Medical Devices.

[9] Rebitschek FG, Gigerenzer G (2020). Assessing the quality of digital health services: how can informed decisions be promoted? [Article in German]. Bundesgesundheitsbl.

[10] Ayers JW, Zhu Z, Poliak A (2023). Evaluating artificial intelligence responses to public health questions. JAMA Netw Open.

[11] Andrikyan W, Sametinger SM, Kosfeld F (2025). Artificial intelligence-powered chatbots in search engines: a cross-sectional study on the quality and risks of drug information for patients. BMJ Qual Saf.

[12] Hung C, Katapally TR (2025). Assessing the role of digital literacy in accessing and utilising virtual healthcare services: a systematic review protocol. J Eval Clin Pract.

[13] Norman CD, Skinner HA (2006). eHEALS: the eHealth Literacy Scale. J Med Internet Res.

[14] Milanti A, Norman C, Chan DNS, So WKW, Skinner H (2025). eHealth Literacy 3.0: updating the Norman and Skinner 2006 model. J Med Internet Res.

[15] Sinha M, Fischer F, Giehl C (2026). Relevance of eHealth Literacy (eHL) for health services research and practice - part I [Article in German]. Gesundheitswesen.

[16] Fitzpatrick PJ (2023). Improving health literacy using the power of digital communications to achieve better health outcomes for patients and practitioners. Front Digit Health.

[17] Busse TS, Nitsche J, Kernebeck S (2022). Approaches to improvement of digital health literacy (eHL) in the context of person-centered care. Int J Environ Res Public Health.

[18] Hua Z, Yuqing S, Qianwen L, Hong C (2025). Factors influencing eHealth literacy worldwide: systematic review and meta-analysis. J Med Internet Res.

[19] Milanti A, Chan DNS, Parut AA, So WKW (2023). Determinants and outcomes of eHealth literacy in healthy adults: a systematic review. PLoS One.

[20] Estrela M, Semedo G, Roque F, Ferreira PL, Herdeiro MT (2023). Sociodemographic determinants of digital health literacy: a systematic review and meta-analysis. Int J Med Inform.

[21] Sainimnuan S, Preedachitkul R, Petchthai P, Paokantarakorn Y, Siriussawakul A, Srinonprasert V (2025). Low prevalence of adequate eHealth literacy and willingness to use telemedicine among older adults: cross-sectional study from a middle-income country. J Med Internet Res.

[22] Jiang X, Wang L, Leng Y (2024). The level of electronic health literacy among older adults: a systematic review and meta-analysis. Arch Public Health.

[23] Heponiemi T, Kainiemi E, Virtanen L (2023). Predicting internet use and digital competence among older adults using performance tests of visual, physical, and cognitive functioning: longitudinal population-based study. J Med Internet Res.

[24] Ge H, Li J, Hu H, Feng T, Wu X (2025). Digital exclusion in older adults: a scoping review. Int J Nurs Stud.

[25] Clegg A, Young J, Iliffe S, Rikkert MO, Rockwood K (2013). Frailty in elderly people. Lancet.

[26] Hoogendijk EO, Afilalo J, Ensrud KE, Kowal P, Onder G, Fried LP (2019). Frailty: implications for clinical practice and public health. Lancet.

[27] Reissmann M, Oswald V, Zank S, Tesch-Römer C, Kaspar R, Simonson J, Tesch-Römer C, Wagner M, Zank S (2023). Old Age in Germany Studies on Health and Society, Vol 8 Springer, Berlin, Heidelberg.

[28] Çöme O, Gökdemir Ö, Bilik Sezer B, Kasapoğlu SS, Kjær NK, Güldal D (2025). The interplay between cognitive function and digital health literacy among older adults: implications for e-health equity and accessibility. Int J Med Inform.

[29] Azizan A (2026). Barriers and motivators of geriatric engagement in digital therapeutics: a narrative review. Ageing Res Rev.

[30] Aslan A, Mold F, van Marwijk H, Armes J (2024). What are the determinants of older people adopting communicative e-health services: a meta-ethnography. BMC Health Serv Res.

[31] Wilson J, Heinsch M, Betts D, Booth D, Kay-Lambkin F (2021). Barriers and facilitators to the use of e-health by older adults: a scoping review. BMC Public Health.

[32] Sezgin D, Hegerath-Segler FM, Christie H (2025). Recommendations for the development and use of technology to support people living with dementia and caregivers: a Delphi study. Alzheimers Dement.

[33] Cuschieri S (2019). The STROBE guidelines. Saudi J Anaesth.

[34] Chatsatrian M, Kunde K, Bosompem J (2025). Usability evaluation of digital health applications for older people with depressive disorders: prospective observational study in a mixed methods design. JMIR Hum Factors.

[35] Mai A, Pape M, Busse TS (2025). Implementation of a digital nurse to improve the use of digital health applications (DiGA) for older people with depressive disorders (DiGA4Aged): a randomized proof of concept study. Trials.

[36] Giehl C, Chatsatrian M, Vollmar HC (2024). Exploring accessibility, user experience and engagement of digital media among older patients with depression: a pilot and observational screening study protocol of the DiGA4Aged study. BMJ Open.

[37] Marsall M, Engelmann G, Skoda EM, Teufel M, Bäuerle A (2022). Measuring electronic health literacy: development, validation, and test of measurement invariance of a revised German version of the eHealth literacy scale. J Med Internet Res.

[38] Petrič G, Atanasova S, Kamin T (2017). Ill literates or illiterates? Investigating the eHealth literacy of users of online health communities. J Med Internet Res.

[39] Xie L, Zhang S, Xin M, Zhu M, Lu W, Mo PKH (2022). Electronic health literacy and health-related outcomes among older adults: a systematic review. Prev Med.

[40] Huang YQ, Liu L, Goodarzi Z, Watt JA (2023). Diagnostic accuracy of eHealth literacy measurement tools in older adults: a systematic review. BMC Geriatr.

[41] Crocker B, Feng O, Duncan LR (2023). Performance-based measurement of eHealth literacy: systematic scoping review. J Med Internet Res.

[42] Wang C, Chang L, Chen X, Kong J, Qi H (2025). eHealth literacy assessment instruments: scoping review. J Med Internet Res.

[43] Nasreddine ZS, Phillips NA, Bédirian V (2005). The Montreal Cognitive Assessment, MoCA: a brief screening tool for mild cognitive impairment. J Am Geriatr Soc.

[44] Lübke N, Meinck M, von Renteln–Kruse W (2004). The Barthel Index in geriatrics. a context analysis for the Hamburg Classification Manual [Article in German]. Z Gerontol Geriat.

[45] Rockwood K, Song X, MacKnight C (2005). A global clinical measure of fitness and frailty in elderly people. CMAJ.

[46] Singler K, Gosch M, Antwerpen L (2020). Clinical Frailty Scale (CFS) [Report in Dutch]. https://www.dggeriatrie.de/images/Bilder/PosterDownload/200331_DGG_Plakat_A4_Clinical_Frailty_Scale_CFS.pdf.

[47] Heidenblut S, Zank S (2010). Development of a new screening instrument for geriatric depression. The depression in old age scale (DIA-S) [Article in German]. Z Gerontol Geriatr.

[48] Harris PA, Taylor R, Minor BL (2019). The REDCap consortium: building an international community of software platform partners. J Biomed Inform.

[49] Jørgensen BB, Gregersen M, Pallesen SH, Damsgaard EMS (2023). Computer habits and digital literacy in geriatric patients: a survey. Digit Health.

[50] Arcury TA, Sandberg JC, Melius KP (2020). Older adult internet use and eHealth literacy. J Appl Gerontol.

[51] Kruger J, Dunning D (1999). Unskilled and unaware of it: how difficulties in recognizing one’s own incompetence lead to inflated self-assessments. J Pers Soc Psychol.

[52] Richardson S, Lawrence K, Schoenthaler AM, Mann D (2022). A framework for digital health equity. NPJ Digit Med.

[53] Hunsaker A, Nguyen MH, Fuchs J, Djukaric T, Hugentobler L, Hargittai E (2019). “He explained it to me and I also did it myself”: how older adults get support with their technology uses. Socius.

